# Supplementary material for: Is Tempranillo Blanco Grapevine Different from Tempranillo Tinto Only in the Color of the Grapes? An Updated Review
Source: Plants (Basel). 2022 Jun 23;11(13):1662. doi: 10.3390/plants11131662 (PMC9269498; doi:10.3390/plants11131662)
Supplement: Supplementary file 1 [file plants-11-01662-s001.zip › SupplFigures_Kizildenizetal_Plants2022_Proofs.pptx]

## Slide 1
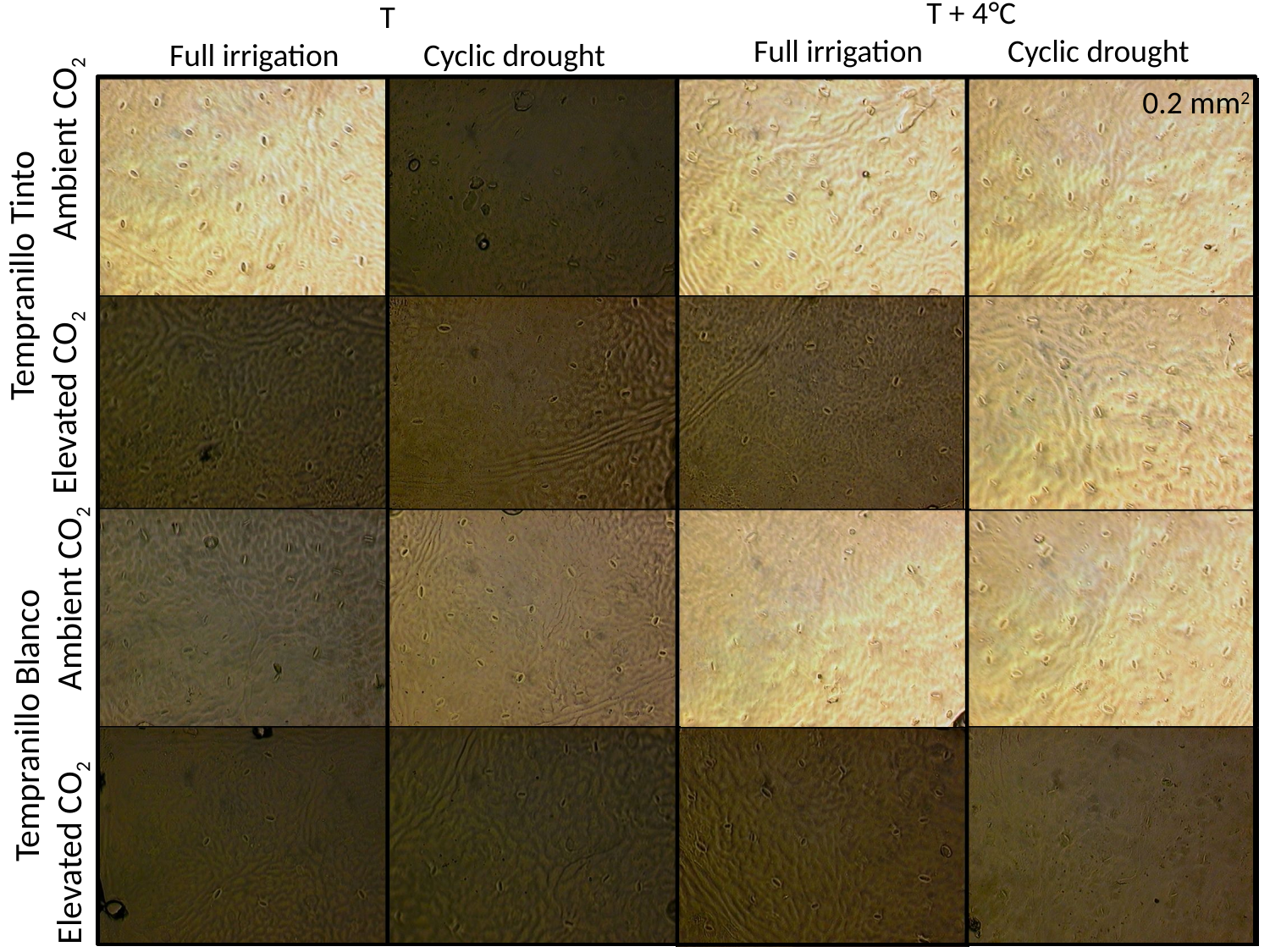

T + 4°C
Full irrigation	Cyclic drought
T
Full irrigation	Cyclic drought
0.2 mm2
Tempranillo Tinto
Elevated CO2	Ambient CO2
Tempranillo Blanco
Elevated CO2	Ambient CO2

## Slide 2
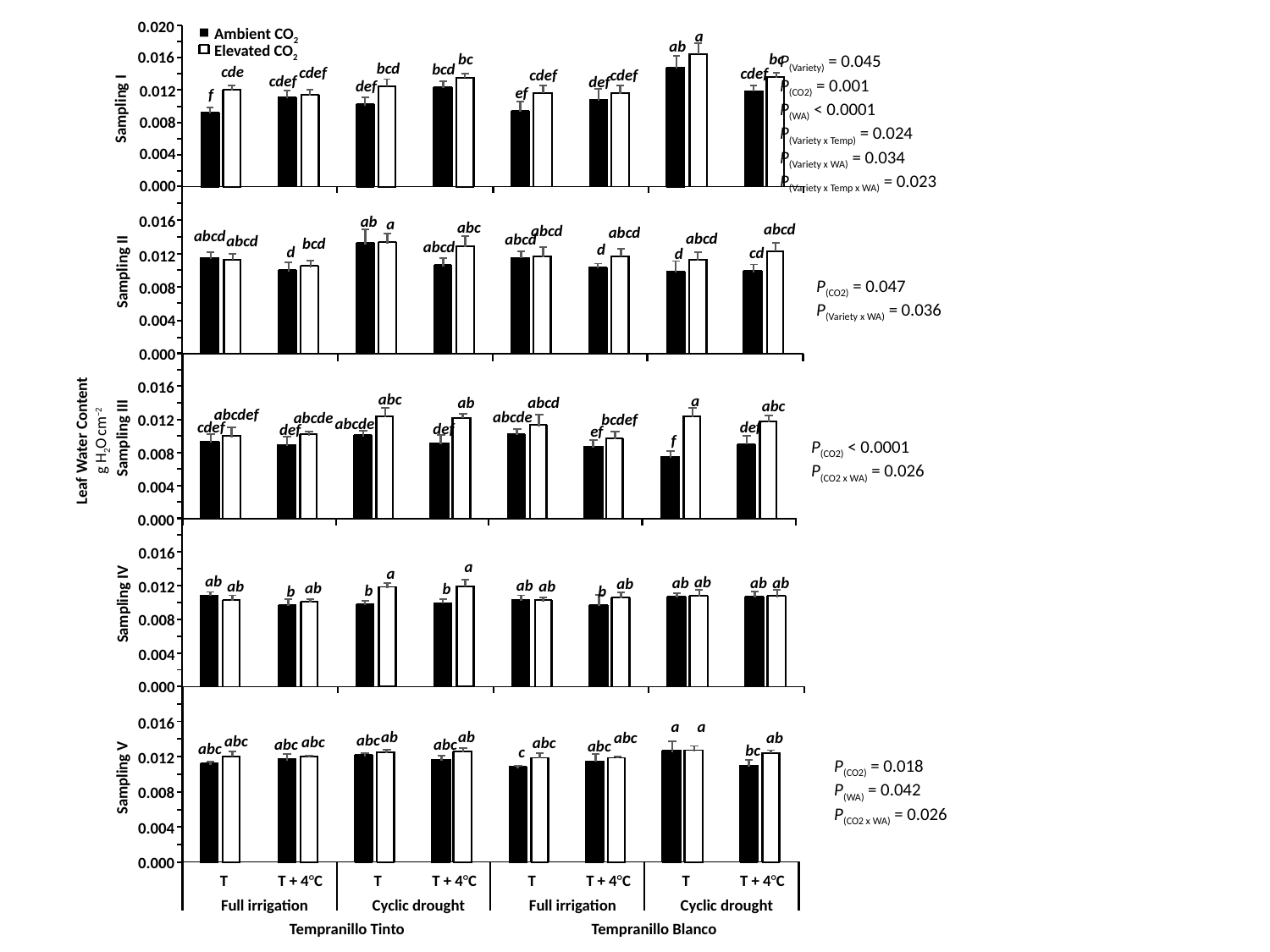

0.020
a
ab
0.016
bc
bc
bcd
bcd
cde
cdef
cdef
cdef
cdef
cdef
def
def
0.012
ef
f
0.008
0.004
0.000
0.016
ab
a
abc
abcd
abcd
abcd
abcd
abcd
abcd
abcd
bcd
abcd
d
d
cd
d
0.012
0.008
0.004
0.000
0.016
abc
a
abcd
ab
abc
abcdef
abcde
abcde
bcdef
0.012
abcde
cdef
def
def
def
ef
f
0.008
0.004
0.000
Ambient CO2
Elevated CO2
Sampling I
Sampling II
Leaf Water Content
g H2O cm−2
Sampling III
0.016
a
a
ab
ab
ab
ab
ab
ab
ab
ab
ab
0.012
ab
b
b
b
b
0.008
0.004
0.000
Sampling IV
Sampling V
0.016
a
a
ab
ab
ab
abc
abc
abc
abc
abc
abc
abc
abc
abc
bc
c
0.012
0.008
0.004
0.000
T
T + 4°C
T
T + 4°C
T
T + 4°C
T
T + 4°C
Full irrigation
Cyclic drought
Full irrigation
Cyclic drought
Tempranillo Tinto
Tempranillo Blanco
P(Variety) = 0.045
P(CO2) = 0.001
P(WA) < 0.0001
P(Variety x Temp) = 0.024
P(Variety x WA) = 0.034
P(Variety x Temp x WA) = 0.023
P(CO2) = 0.047
P(Variety x WA) = 0.036
P(CO2) < 0.0001
P(CO2 x WA) = 0.026
P(CO2) = 0.018
P(WA) = 0.042
P(CO2 x WA) = 0.026

## Slide 3
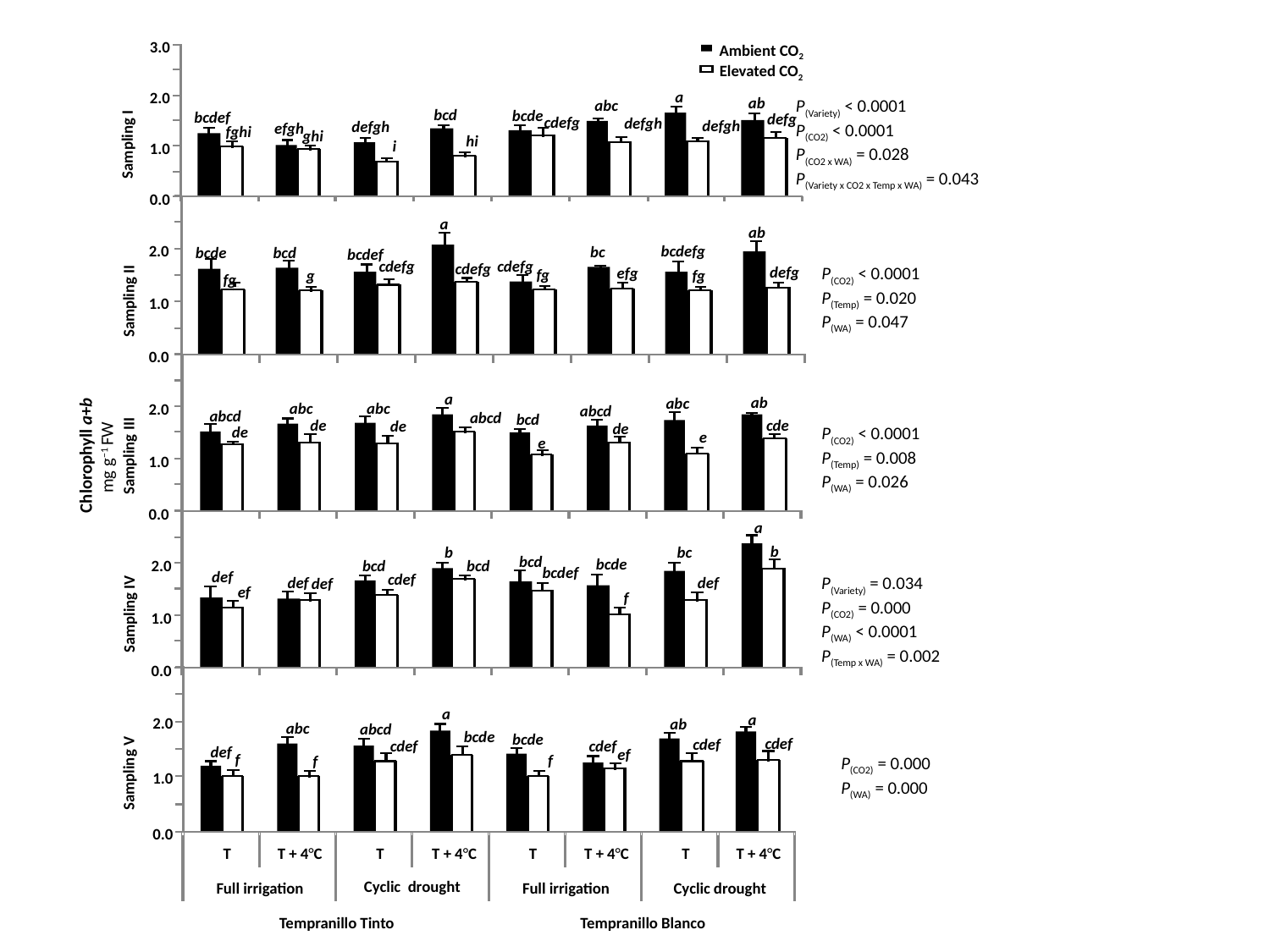

3.0
Ambient CO2
Elevated CO2
a
2.0
ab
abc
bcd
bcde
bcdef
defg
cdefg
defgh
defgh
defgh
efgh
fghi
ghi
hi
i
1.0
0.0
a
ab
bcdefg
2.0
bc
bcd
bcde
bcdef
cdefg
cdefg
cdefg
defg
efg
fg
g
fg
fg
1.0
0.0
a
ab
abc
abc
abc
2.0
abcd
abcd
abcd
bcd
de
cde
de
de
de
e
e
1.0
0.0
a
b
b
bc
bcd
bcde
bcd
bcd
2.0
bcdef
def
cdef
def
def
def
ef
f
1.0
0.0
a
a
2.0
ab
abc
abcd
bcde
bcde
cdef
cdef
cdef
cdef
def
ef
f
f
f
1.0
0.0
T
T + 4°C
T
T + 4°C
T
T + 4°C
T
T + 4°C
Cyclic drought
Full irrigation
Full irrigation
Cyclic drought
Tempranillo Tinto
Tempranillo Blanco
Sampling I
Sampling II
Chlorophyll a+b
mg g−1 FW
Sampling III
Sampling IV
Sampling V
P(Variety) < 0.0001
P(CO2) < 0.0001
P(CO2 x WA) = 0.028
P(Variety x CO2 x Temp x WA) = 0.043
P(CO2) < 0.0001
P(Temp) = 0.020
P(WA) = 0.047
P(CO2) < 0.0001
P(Temp) = 0.008
P(WA) = 0.026
P(Variety) = 0.034
P(CO2) = 0.000
P(WA) < 0.0001
P(Temp x WA) = 0.002
P(CO2) = 0.000
P(WA) = 0.000

## Slide 4
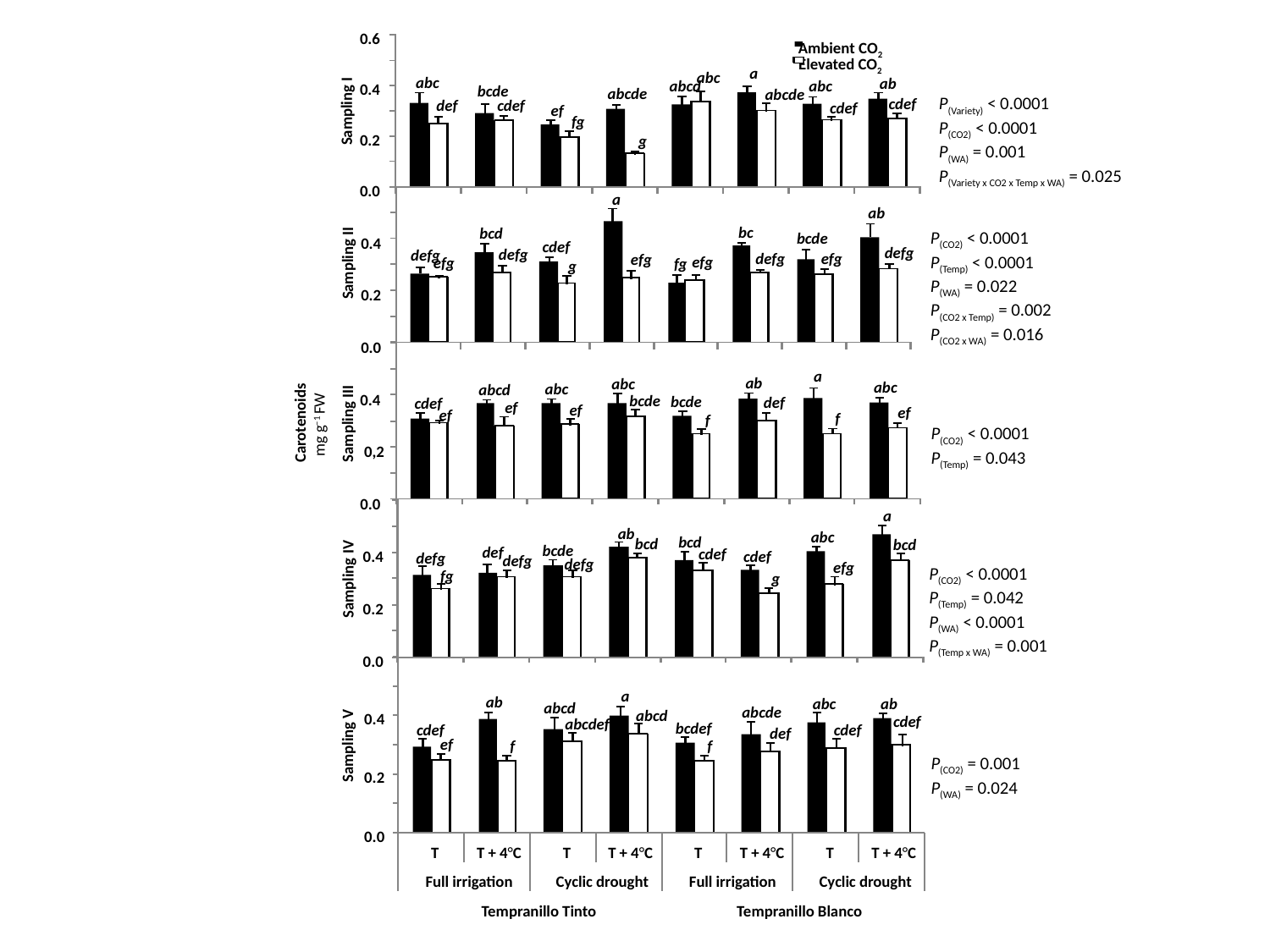

0.6
Ambient CO2
Elevated CO2
a
abc
abc
ab
abcd
abc
0.4
bcde
abcde
abcde
cdef
def
cdef
cdef
ef
fg
0.2
g
0.0
a
ab
bc
bcd
bcde
0.4
cdef
defg
defg
defg
defg
efg
efg
efg
efg
fg
g
0.2
0.0
a
ab
abc
abc
abc
abcd
0.4
bcde
bcde
def
cdef
ef
ef
ef
ef
f
f
0,2
0.0
a
ab
abc
bcd
bcd
bcd
bcde
def
cdef
0.4
cdef
defg
defg
defg
efg
fg
g
0.2
0.0
a
ab
abc
ab
abcd
abcde
abcd
0.4
cdef
abcdef
bcdef
cdef
cdef
def
ef
f
f
0.2
0.0
T
T + 4°C
T
T + 4°C
T
T + 4°C
T
T + 4°C
Full irrigation
Cyclic drought
Full irrigation
Cyclic drought
Tempranillo Tinto
Tempranillo Blanco
Sampling I
Sampling II
Carotenoids
mg g−1 FW
Sampling III
Sampling IV
Sampling V
P(Variety) < 0.0001
P(CO2) < 0.0001
P(WA) = 0.001
P(Variety x CO2 x Temp x WA) = 0.025
P(CO2) < 0.0001
P(Temp) < 0.0001
P(WA) = 0.022
P(CO2 x Temp) = 0.002
P(CO2 x WA) = 0.016
P(CO2) < 0.0001
P(Temp) = 0.043
P(CO2) < 0.0001
P(Temp) = 0.042
P(WA) < 0.0001
P(Temp x WA) = 0.001
P(CO2) = 0.001
P(WA) = 0.024
