# Supplementary material for: Is Tempranillo Blanco Grapevine Different from Tempranillo Tinto Only in the Color of the Grapes? An Updated Review
Source: Plants (Basel). 2022 Jun 23;11(13):1662. doi: 10.3390/plants11131662 (PMC9269498; doi:10.3390/plants11131662)
Supplement: Supplementary file 1 [file plants-11-01662-s001.zip › Table S1.pdf]

Table S1. Total anthocyanins (Anth) at maturity in Tempranillo Tinto grapevine (*Vitis vinifera* L.) plants grown under different CO_2 concentrations (ambient (400 ppm) (ACO_2) or elevated (700 ppm) (ECO_2)), temperature regimes (ambient (T) or ambient + 4 °C (T+4°C)) and water availability (full irrigation (FI) or cyclic drought (CD)). Different letters indicate significant differences among treatments (P < 0.05) based on LSD test (n = 3, mean ± SE). Means without letters or with common letters are not statistically different. Only main factors and interactions P-values that were significant are shown. Data were obtained by T. Kizildeniz during her PhD Thesis following the procedure described in Acevedo De la Cruz et al. [37].

			**Anth (mg g^{-1} Skin DW)**	**Anth (mg g^{-1} Skin DW)**		
			2013-2015	2013	2014	2015
T	ACO_2	FI	29.6 ± 3.2	25.9 ± 4.1 ab	26.8 ± 3.0 abc	36.0 ± 2.2 a
		CD	26.3 ± 4.4	28.9 ± 2.3 a	17.7 ± 1.0 d	32.3 ± 1.4 abc
	ECO_2	FI	24.0 ± 2.8	18.6 ± 1.6 bc	27.8 ± 4 ab	25.6 ± 1.0 bc
		CD	22.6 ± 0.6	22.7 ± 2.6 abc	21.6 ± 2.3 bcd	23.5 ± 0.4 c
T+4°C	ACO_2	FI	28.9 ± 3.6	21.9 ± 1.7 abc	31.6 ± 1.2 a	33.2 ± 6.0 ab
		CD	21.1 ± 4.2	14.8 ± 1.0 c	19.4 ± 0.7 d	29.0 ± 4.8 abc
	ECO_2	FI	25.1 ± 3.8	17.7 ± 1.5 c	27.0 ± 2.2 abc	30.5 ± 1.5 abc
		CD	23.0 ± 2.6	20.6 ± 4.3 bc	20.3 ± 1.3 cd	28.2 ± 2.6 abc
CO_2			0.255	0.129	0.858	**0.020**
Temp			0.648	**0.012**	0.499	0.690
Irrigation			0.143	0.687	**< 0.0001**	0.173
CO_2 x Temp			0.442	0.057	0.195	0.093
CO_2 x Irrigation			0.427	0.159	0.198	0.685
Temp x Irrigation			0.591	0.150	0.582	0.933
CO_2 x Temp x Irrigation			0.681	0.250	0.688	0.966
